# 

**Published:** 1892-02

**Authors:** 


					﻿ESTABLISHED 1856.
SUBSCRIPTION, $2.00.
UDITEI) BY
H. V. M. MILLER, M. D., LL. D.
VIRGIL O. 11ARDON, M. D.
F. W. McRAE, M. D.
L.	P. KENNEDY, A. M., M D
M.	B. HUTCHINS, M. D.
L. B. GRANDY, M. D.
Published by JAS. P. HARRISON & tO., Atlanta, Ga.
Entered at the Post-Office at Atlanta, Ga., as second-class matter.
New sTrik Vol. VHL ! ATLANTA, Ga., FEBRUARY, 1892. No , L2.
				

